# The effect of compliance to Hand hygiene during COVID-19 on intestinal parasitic infection and intensity of soil transmitted helminthes, among patients attending general hospital, southern Ethiopia: Observational study

**DOI:** 10.1371/journal.pone.0270378

**Published:** 2022-06-29

**Authors:** Mohammed Seid, Tsegaye Yohanes, Yitagesu Goshu, Kiyar Jemal, Munira Siraj

**Affiliations:** Department of Medical Laboratory Science, College of medicine and Health Sciences, Arba Minch University, Arba Minch, Southern Ethiopia; Abadan University of Medical Sciences, ISLAMIC REPUBLIC OF IRAN

## Abstract

**Background:**

Intestinal parasitic infection (IPIs) is one of the major health problems in Sub -Saharan Africa where water, sanitation and hygiene practices are inadequate. Taking into account the national level implementation of intensive hand hygiene against COVID-19 pandemic and general protective effect this study assessed its effect on intestinal parasite.

**Objective:**

This study aim to investigate the effect of compliance to hand hygiene practice on the prevalence of intestinal parasitic infection (IPIs) and intensity of Soil transmitted helminthes (STH) among patients attending tertiary care hospital in southern Ethiopia.

**Methods:**

Observational study was conducted from June to September 2021. Data on socio demographic, hand hygiene practice and intestinal parasite (prevalence and intensity of helminthic infection) was collected from randomly selected and consented patients. Compliance to hand hygiene practice was assessed using pre-tested questionnaire. Fresh stool sample from each participant was examined by direct wet mount, concentration and Ziehl-Neelson (ZN) staining technique to detect intestinal parasite. Intensity of STH measurements was done through direct egg-count per gram using Kato Katz methods. Data analysis was done using SPSS version 25. Odds ratio with 95% confidence interval was used to measure association and p-value <0.05 was considered as statistically significant.

**Results:**

The study population (N = 264) consisted of 139(52.65%) male and 125 (47.34%) female with the mean ages of 36 ±16.12(±SD). The proportion of good compliance to hand hygiene during COVID-19 to was 43.93% (95%CI: 37% to 47) and prevalence of intestinal parasite was 26.14% (95%CI:21.2% to 31.75) comprising 23.48% intestinal protozoa and 6.43% of soil transmitted helminthic infection. *Gardia lamblia*, *Entamoeba histolytica/dispar*, *Ascaris lumbricoides* were the common parasite in the study area with prevalence of 15.53%, 6.44%, and 1.52% respectively. Prevalence of intestinal parasite among participants with good compliance to hand hygiene group and poor compliance to hand hygiene were (14.65% vs. 35.13%)(AOR: 0.48,95%CI:0.13 to 0.68) (p = 0.002) implying that good compliance to hand hygiene can reduce the risk of IPIs by 52%. Moreover significantly lower odds of intestinal protozoa among good compliance to hand hygiene group than the control (OR:0.38; (95%CI: 0.20 to 0.71);P = 0.001. However, no significant difference in the odds of intensity of STH infection in good compliance hand hygiene and poor compliance group. The result of this study also confirmed the association between intestinal parasitic infections and younger /adolescent age, education status, habit of eating raw vegetable and figure nail status.

**Conclusion:**

Good hand hygiene compliance during COVID-19 significantly associated with reduction of intestinal parasitic infection. This finding highlights the secondary protective effect of improved hand hygiene against IPIs and suggest it can used in augmenting the existing parasitic control strategies in the study setting.

## Introduction

Intestinal parasitic infections (IPIs) caused by intestinal helminthes and protozoan parasites and are the most widespread health maladies distributed throughout the world today. Intestinal parasitic infections are on the world health organization’s list of neglected tropical disease (NTDs [1, 2] and often result high burden of morbidity, and socio economic crisis in low and middle income countries. Current estimates suggest that at least one-quarter of the world’s population is infected with intestinal parasites where 450 million individuals developed the diseases [2]. The burden of disease due to these intestinal parasites resulting in an estimated 4.98 years of life lost due to disability (YLDs) and 5.18 million disability-adjusted life years (DALYs). IPIs disproportionately affected community in the tropics and subtropics region with the greatest in sub-Sahara Africa, South Asia, and South America [3–5]. Low socio-economic status, water, sanitation and hygiene (WASH) and climatic conditions are the common reason for maintain intestinal parasitic infections to be an endemic disease in tropic and sub-tropical region. Given that most of the common intestinal parasitic infections of human are acquired through feaco oral route of transmission either directly hand-to-mouth or indirectly through food and water, WASH and mass drug administration (MDA) is regarded as an effective intervention for interrupting person-to person transmission to control the problem [6].

The type and distribution of intestinal parasitic infection (IPIs) varied in different parts of the world. The public health concern intestinal helminthic parasites, also known as geohelminths and soil-transmitted helminths (STH) in tropical and subtropical regions of the developing world are *Ascaris lumbricoides (A*. *lumbricoides)*, *Trichuris trichiura (T*. *trichiura)*, *Ancylostoma duodenale*, and *Necator americanicus* (hookworms). STH still infected an estimated one billion people globally and account approximately 85% of the neglected tropical diseases (NTDs) burden [1, 3]. The most common intestinal protozoan parasites are: *Giardia intestinalis*, *Entamoeba histolytica*, and *Cryptosporidium parvum* [7]. Intestinal parasites rarely caused death but the serious health impact is associated with children who had repeated infection which may leads to a heavy parasite infestation of the gut. A recent review by Abedi H.S *et*.*al* concluded that intestinal protozoa infection ultimately end up with chronic infections that can bring serious complications such as irritable bowel syndrome, prolonged diarrhea, stunting of physical growth and slowing of mental development [8]. Infection with intestinal helminthes notable cause a wide range of immune modulation which may have an effect on the person’s ability to cope with subsequent infection [6, 9]. Trend in prevalence of soil transmitted infection (STH) showed a sharp decline in the past decade in few countries but infection and its consequence remains big on the agenda that require further actions in many developing country [10].

Scientific evidence suggests that hand hygiene as one of the most effective measures to control infection [11]. For example a recent Cochrane review and meta-analysis found sufficient evidence to conclude hand hygiene practice as cost effective control measure in reducing gastrointestinal and respiratory tract infection [3, 12, 13]. Theoretically, hand hygiene to prevent IPIs could require washing/ sanitizing of hand using water and soap (anti-bacterial, non-anti-bacterial soap) or with waterless hand sanitizer (alcohol based hand sanitizer),on regular basis, at appropriate critical time, for correct time and following standard step by step procedure [14]. The evidence on the literature also indicate hand hygiene intervention for decreasing infection achieved by mechanical removal of disease‐causing organisms; and or chemically killing both at home and community setting [15–18]. The effect size of hand hygiene on reduction of infection varied in different setting and the species of pathogens. For example a systematic reviews, in which result from randomized control trial (RCT)were pooled concluded 31% (95% CI: 19 to42%) reduction of gastrointestinal infection (ranging from 0%-70% [19]. A separate paper by Freeman MC *et*.*al* showed the association of hand hygiene and 27% lower odds of *A*. *lumbricoides* infection, 20% reduction of *T*. *trichuria* and 35% reduction of Hookworm [20].

Despite good compliance to hand hygiene is seen as the most important method to prevent the transmission of gastro intestinal infection, the adoption and adherence to hand hygiene in the war against IPIs seems very poor. Effort in up scaling of the most common benefit of this intervention for improving the global health faced challenged by multiple factors. Lack of access to WASH facility, compliance to hand hygiene(HH), poor socio -economic status are some of the factors that makes the success rate from this public intervention varied from place to place. Moreover available evidence that seemingly showed the convincing direct and indirect health benefit of improved hand hygiene, should be interpreted cautiously by considering uncertainty related with methodological flaws, such as different in adherence to hand hygiene practices, different study population, uncontrolled confounding, failure to include an appropriate control group, different study setting and biases in accurately measuring hand washing behaviors [15, 19, 20]. Nevertheless compliance to hand hygiene has been linked with the effectiveness of the intervention in reducing transmission of pathogenic microorganisms which is even more relevant in the current coronavirus disease 2019 (COVID-19) pandemic [11, 18, 21, 22]. Currently WHO/ Centers for Disease Control and Prevention (CDC) and Ethiopian minster of health jointly released statement of recommendation on intensive hand hygiene as one of the essential means to prevent the spread of COVID-19 [23–25]. Unfortunately, overall, hand hygiene compliance remains insufficient, and compliance levels as low as 5% have been reported from low-income countries [26]. This calls for further research to assess the compliance and to uncover the additional impact added by this public measures. Hence, this study is designed to assess compliance to HH during acute phase of COVID-19 and its effect on intestinal parasite (prevalence and intensity) in southern Ethiopia. The finding may provide new insights into the secondary benefit of hand hygiene in the control of IPIs and may inform the public health and policy maker to promote hand hygiene in post COVID-19 pandemic.

## Materials and methods

### Study design and setting

Cross-sectional observational study was conducted from June to September 2021at ArbaMinch General Hospital. This hospital is 350 bed governmental hospitals situated, in ArbaMinch town, Gamo zone, Southern Nation and Nationalities peoples region (SNNPR) of southern Ethiopia. Arba Minch General Hospital is the hospital currently serving as a referral centre for tertiary specialist care for a catchment population of approximately 3 million people from Gamo zone, Gofa Zones & other Zuria wereda. The total population of Gamo Zone is approximately 1659310. While 157,446 or 9.88% are urban inhabitants, a further 480 or 0.03% are pastoralists. Arba Minch town is the administrative town of in Gamo zone, located about 500 km south from Addis Ababa (the capital of the Ethiopia) at an elevation of 1285meters above sea level and 275 km from Hawassa, the capital of the SNNPR having a total Area of 28 km2, latitude and longitude of 11°36′N 37°23′E and an elevation of 1840m (6,040 ft. feet) above sea level. Currently, Gamo zone had five governmental hospitals (in Arba Minch town, 2 in hospitals, two health centers and different private profitable and non-profitable health institutions [27].

### Population

The study population was patients attending outpatient department of Arba Minch General Hospital during study period. Study group: Good hand hygiene compliance during COVID -19 Comparison group or Control group: Poor hand hygiene compliance during COVID -19.

#### Eligibility criteria

Only respondents who were age above 6 years and above, identified themselves as living in one of the district of Gamo Zone and volunteer to participate are included in the study. Excluded from the study were those who took anti-helminthic drug/antiprotozoal within the past two week prior to the study, handicapped, severely ill, healthcare workers and unable to provide the stool sample.

### Sample size/sampling methods

We assumed a null hypothesis of no change in the prevalence of IPIs as assessed in a cross-sectional survey of patients of Jimma before COVID-19 pandemic when it was estimated at 20.6% [6]. In order to detect at least a 10% decline in prevalence (from 20% to 10.6%) at the 95% of level of confidence, 5% level of significance and power of 80%, stool specimens from 251 patients needed to be examined. The sample size for the present study is determined by using single population. Then by considering 5% for non-response rate the total sample size became 264. Systematic random sampling technique was applied to select the study participants. K value was determined by considering the number of patients who visited the out patients department of Arba Minch hospitals in the previous year in period similar to the study period. A total of 2378 patients were found from the record of health information management system. Then K value was obtained by dividing 2387 by sample size (264), K = 9. A sampling interval of 9 was achieved. The first participant was selected using a lottery method between number 1 and 9 thereafter a sampling interval of 5 was used for the subsequent patients that present themselves at the facility until the sample size was achieved. If the Kth participant was not convenient, the next was sampled.

### Measurement of outcomes and definition

The primary outcomes were prevalence of intestinal parasitic infection. This was defined as the detection of one or more intestinal parasite (protozoa and/ helminthes) in fresh stool specimen by microscopic techniques [6]. The secondary outcome was the intensity of soil transmitted helminthic infection. It was determined by directly counting helminthes eggs excreted in faeces (expressed as eggs per gram, EPG). Infection intensity of the STHs was estimated by multiplying the total number of eggs counted by 24, which gives as the eggs per gram (EPG) of stool. Besides, the species-specific classes of infection intensity were classified as light, moderate and heavy as per the threshold set by WHO [28].

### Assessment of compliance to hand hygiene and ascertainment

Compliance to Hand hygiene was evaluated by using checklist according as the WHO and CDC recommendation [21, 23, 29]. Hand hygiene was defined as the cleansing of hands with soap and water or with antiseptic hand rub (alcohol-based hand sanitizer). Criteria of compliance: According to the process recommended by the hand hygiene (HH) guidelines, the criteria for compliance included critical timing for hand hygiene practice (indication), procedure, duration, frequency, hand hygiene method, and the overall for all criteria. Compliance assessment was done using questionnaire which include five questions about the critical timing for hand hygiene, four question on hand washing methods (selection), three question on the frequency, and three question on duration of hand hygiene, and two hand hygiene steps demonstrated. Therefore, hand hygiene practices were measured using 17 questions (Cronbach’s alpha 0.79) where respondents were examined on a five-point Likert scale in which the participants scored choose from- always, often, sometimes, rarely and never. In the evaluation of ‘always’ response received 5 points, often received four “sometimes” received three points, “rarely” received two point and “never received one point for all items. Finally participants were asked to demonstrate all steps of hand hygiene practice and those who demonstrated five and more than five steps of hand hygiene (HH) procedure was received five points, 4–3 key steps received ‘4 points, 4 to 3 steps but in disordered fashioned received 3, observation with less than three key steps get two, one or no were relieved one point. Overall individual compliance percentage score to hand hygiene was calculated by taking the average of participants scores at each section such as critical time, hand washing methods, frequency and duration of hand washing and hand hygiene steps demonstrated. After one questionnaires were excluded due to incomplete filling and non-compliance of inclusive criteria the total score for hand hygiene was calculated as (16 x 5 = 80). Study participants hand hygiene practice was dichotomized into “good compliance “as exposed group and “poor compliance” as comparison group based on the cut off values to determine good, and poor levels which were adapted from previously published study [14, 30]. Those participants who scored mean and above mean of the overall questions (total score = 80) were categorized in good compliance to hand hygiene practice and poor compliance to hand hygiene practice. Accordingly of the total 264 study participants screened for their adherence to hand hygiene, (n = 116) participants were found to have good score in compliance to hand hygiene and enrolled as study group whereas those who had poor score in compliance to hand hygiene (n = 148) were recruited in the study as control group.

### Potential confounders

Data was collected on other risk factors for intestinal parasite infection and the estimates of the effect of exposure on infection adjusted for potential confounding factors. As potential confounders we considered demography and hygiene related factors such as age, sex, education and socioeconomic status, residence, habit of consuming raw meat and vegetables, finger nail status, shoe wearing habit, open filed defecation,(II) water related such as source of water, habit of treating water and environmental factors such as availability of latrine.

### Data collection instrument and tools

A semi-structured questionnaire, observational checklist/ data abstraction form were used to gather maximum information. The data collection tools were developed based on comprehensive literature review, and the questions were built based on relevant reviewed literature with some modifications based on contextual situation. Three self-reported questionnaires, adapted from previously published studies were utilized for this study [14, 31]. The questionnaire contained multiple questions divided into three main sections; part I (socio-demographic characteristics such as sex age, place of residence, educational level, socioeconomic status, occupations), part II (compliance to hand hygiene practice) part III (water & sanitation such as water source, availability of toilets). A summary of the questions and the scale used for measurement is included in (S1 Table).

The questionnaire was prepared originally in English according to the research objectives and possible associated factors in the local situation and then translated in to the local language. To ensure the reliability of the information given during data collection first pilot study was done in Shelle Health center and the patients were interviewed in their mother tongue. Then selected patients were asked using the revised and pretested structured questionnaires.

### Laboratory methods and detection of intestinal parasite

#### Stool specimen collection and processing

A single fresh stool specimen was collected from each study participant. After instructing each study participant about fresh stool specimen collection, a dry, clean, leak-proof, detergent free and labeled stool cup was delivered to bring a fresh stool of 5-10g. If the patient has diarrhea respondents were instructed to put about a teaspoonful amount of stool into leak-proof wide-neck stool containers. Immediately after collection, some portion of the stool samples were examined for intestinal parasites using the standard routine methods (macroscopic, microscopic (direct wet mount, iodine stain) used by the hospitals. The remaining portion was preserved in 10% formalin or sodium acetate acetic acid formalin (SAF) solution and transported to Arba Minch University microbiological & parasitology laboratories where further test such as concentration technique and Kato- Katz technique was done [32].

#### Macroscopic and microscopic examination

The stool samples were observed for color, consistency, presence of blood and mucus, presence of adult worms and segments and other abnormalities.

*Direct wet mount analysis*. A direct saline and iodine wet mount of each sample were performed to detect cysts, oocysts, eggs and larvae trophozoite, of intestinal parasites microscopically according to laboratory practice A direct wet mount was prepared by emulsifying approximately 1-2g of stool using a drop of physiological saline(.85% or Lugol’s iodine solution on a slide. The wet mounts were performed at the site of collection within 30 minutes of sample receipt & samples were examined under a light microscope (Olympus Optical Co., Ltd, Japan) at 100× and 400× magnifcations for possible detections of parasites. Iodine direct smear allows the examination of the characteristic features of the protozoa and the identification of the *Entamoeba histolytica/dispar* cyst from the commensal *Entamoeba coli* [32].

*Formal-ether concentration technique*. A portion of each preserved stool specimen was taken and processed following standard procedures to determine the intensity of helminthes infection according to. Briefly, 1-2g of stool was placed in a clean conical centrifuge tube containing 7 ml 10% formol water by using applicator stick. The resulting suspension was filtered through a sieve into another conical tube. After adding 3–4 ml of diethyl ether to the formalin solution, the content was centrifuged at 3200 rpm for 1 min. The supernatant was discarded; smear was prepared from the sediment and observed under a light microscope for cysts, oocysts, eggs and larvae of intestinal parasites [32].

*Kato-katz technique*. Kato- Katz technique was performed for microscopic examination of intestinal helminths and parasite intensity determination by enumerating ova of *A*. *lumbricoides*, hookworm and *T*. *trichiura*. This was done by taking 1 grams of stool to determine the intensity of the infection in terms of eggs per gram of stool (epg) on each slide. Two slides were prepared from each stool sample and enumerated within 30 minutes of slide preparation. The number of eggs for each helminth parasite detected was counted and multiplied by 12 to obtain the number of eggs per gram of feces. Ten percent subsamples of stool smears were reexamined for quality control purposes. Intensity of infection was categorized as moderate/heavy based on WHO categories (≥5,000 epg for *A*. *lumbricoides*, ≥1,000 epg for hookworm, and ≥2,000 epg for *T*. *trichiura)* [28, 33].

*Modified acid fast staining techniques*. For coccidian parasites, the permanent stained smears using modified Ziehl-Neelsen (ZN) were performed. Smears are prepared after concentration, air dried and then fixed in methanol, stained with Kinyoun carbol-fuchsin for 4–5 minutes, distained with 1% aqueous sulfuric acid for 2–3 minutes, rinsed with distilled water and then counterstained with methylene blue for 1 minute. Smears are rinsed with distilled water drained and dried [32].

### Data management and statistical analysis

Data was checked for completeness and cleaned of any inconsistencies. Data were double entered into EpiData version 3.1 software (Centers for Disease Control and Prevention, Atlanta, 2008) and exported to SPSS version 25 (IBM-SPSS, Inc., Chicago, IL, USA) software for analysis. Cleaning was made to avoid missing values, outliers and other inconsistencies. Consistency and completeness of data were checked using frequency and a 2 by 2 tables. For the descriptive data, frequencies and percentages were used. The Pearson’s Chi-square (Chi2) test and the Fisher’s exact test were used for comparison of proportions of IPIs between different subgroups. Data are presented using tables and graphs. Missing data were managed by observing cross tabulation results and percentages. To assess the effect of compliance to Hand hygiene practice on IPIs, the participants were divided into two groups based on adherence to standard hand hygiene. Binary and multivariable analyses (logistic regression) were performed, considering the detection of any intestinal parasite as dependent variables. Binary logistic regression was used to see the crude association between dependent and explanatory variables and multivariable logistic regression was used to adjust for confounders. Variables with associations generating p value ≤ 0.25 were selected to the multi variable model. Odds ratio with 95% confidence interval was used to measure the strength of association and p-value<0.05 was considered statistically significant at 95% CI. All known and potential confounders (age, income, educational level, occupation, finger nail status, water source, habit of shoe wearing, habit of eating uncooked food availability of laterite, and utilization of laterite) were adjusted for to obtain regression coefficients with robust variance estimates to allow for compliance to Hand washing practice clustering. ANOVA, Independent t-test were used to compare the mean intestinal parasitic egg count between the study and control group.

### Data quality assurance

Initially, the study tool (questionnaire) was prepared in the English language. Then, it was converted into the Amharic language. Lastly, it was retranslated back to English to retain its accuracy and consistency. Prior to data collection, training was given for data collectors about how to collect, process, examine the specimen and how to collect risk factor data. All reagent preparation and stool examination was done following standard operating procedure, analytic, pre analytic and post analytic phase. Stool specimens were checked for their quantity whereas old fecal sample or sample contaminated with soil and urine were discarded and recollected. To eliminate observer bias in egg count, more than one slide from the same sample was prepared and each slide was examined by two experience laboratory technologists (a senior parasitologist). For those cases with discordant results, the principal investigator repeated the test. The results of the principal investigator were considered as the final result.

### Ethical consideration

This study was approved by the IRB of AMU, license number (Ref; AMU /IRB /C /S/ C/ 31 /11 /2021). Letter of support was obtained from Arba Minch General Hospital. Study participants/ care givers were informed about the purpose of the study, benefits and rights. Written and oral consent form was sought from each participant. Signature (or thumbprint, if illiterate) of the participants and parents/guardian of a child was obtained before their enrollment in the study. Identification numbers instead of names of the respondents were used during the research and the data collected were treated with the utmost confidentiality. Participants was informed that they are free to withdraw from the study if they are not willing to participate. In order to protect the confidentiality of information Name and other identification is not include in questioners and to maximum effort was carried out to maintain privacy of the respondent during the interview. Participants found to be positive for intestinal parasites were communicated with clinicians & patients were treated with anti-parasitic drugs as recommended by the World Health Organization

## Results

### Baseline characteristics of study participates

A total of 116 peoples who had a good compliance to the hand hygiene procedure of COVID-19 and 148 who had poor hand hygiene compliance were participated in this study with 95.11 and 100% response rate for each group respectively. The overall the male to female sex ratio (M/F) was 1: 1.1 with 139 male and 125 female. The ages of the study participants ranges from 6–81 with a mean age of (36 ±16.12 SD). Of the five category in age the majority of participants 99(37.50%) were in the age brackets of 15–29 years while children less than 15 years were least age group in proportion 4.9% (n = 13). Participants who attended formal education at different level account 79.54% (n = 210) but 20.45% (n = 43) participants were illiterate. Participants who attended secondary level education (de 9–10) & those attended college/university level education were equally account, 25.75% (n = 68). The participants distribution by their principal occupations showed agriculturist 13.63% (n = 36), daily laborer in 24.2%, and employed (18.6%) but student took the highest proportion 26.89% (n = 71). More than half of participants 66.67% (n = 176) were urban dwellers, and 40.90% (n = 108) of respondent earn monthly income of less than 1000 ETH Birr.

Regarding water sanitation and hygiene (WASH) characteristic in this study about 89.39% of study participants obtain water from protected source, 90.90% of participant trimmed their finger nail regularly, 64.01% respondent wear their shoe regularly and only 19.31% had habit of treating water. Large majority of respondents had laterite at their compound 246(93.70%), but utilization was 161(61.00). A habit of eating raw meat and eating raw vegetable /or unwashed fruit were reported by 81(30.68%) and 119(45.07%) of the participant respectively (S1 Table). Regarding the hand hygiene characteristic, the large majority (92.04%) reported good hand hygiene practice before and after meal followed by before, during and after handling of food (65%), and 65% after defection (toilet). The proportion of participant who wash their hand at higher frequency or more than 6 times a day was 60.97% whereas those who washed 3–5 times/days and less than 2times per day was 28.03% and 34.09% respectively. Water and soap was the most frequently used hand hygiene methods in the area where 62% of participant reportedly used to clean hand. In addition 13% and 10% of the population used water alone and alcohol based antiseptic hand sanitizer alone respectively while the rest reported to use mixed of each (S2 Table).

#### Compliance to hand hygiene

The overall mean hand hygiene score over -one and half year of promotion program was 56.5 ± 11.5SD), with the minimum and maximum scores being 15 and 75, respectively. In this study the overall good compliance to HH was 43.94% (n = 116), 95%CI: 37% to 47).whereas poor hand hygiene practice was 56.06% (n = 148). Individually there were no significant differences in the hand hygiene practice in different age categories. Similarly compliance to hand hygiene was not significant different for different level of education, occupation and monthly income. However females had higher rate of good compliance to hand hygiene compared with males (p<0,001). Surprisingly good compliance to hand hygiene was greater for urban dwellers than peoples from rural area (Table 1).

**Table 1:** Socio-demographic characteristics of study participants stratified by the two group based on the compliance to hand hygiene practice during COVID 19 pandemic in Arba Minch general hospital, southern Ethiopia between June to September 2021 (n = 264).

Variables/ Categories	Frequency (%)	Hand washing practice (observations		
		Good(n = 116)	Poor(n = 148)	p-value
**Response rate** 264(100)				
**Overall score, mean±SD (range)**				
**Age(years)**				
6–14	13(4.92)	12(25.86)	1(0.68)	0.47
15–29	99(37.50)	30(25.86)	69(46.62)	
30–44	63(23.86)	27(23.28)	36(24.32)	
45–59	46(17.42)	19(16.38)	27(18.24)	
>60	43(16.28)	28(24.14)	15(10.14)	
**Gender**				
Male	139(52.30)	44(39.66)	95(64.19)	0.001
Female	125(47.34)	72(60.34)	53(35.81)	
**Residence**				
Urban	176(66.67)	107(60.79)	69(39.20)	0.001
Rural	88(33.33)	35(39.71)	53(60.22)	
**Educational Status**				
Illiterate/read and write	54(20.45)	9(7.76)	45(30.41)	0.58
Primary (grade1-8)grade	42(15.90)	10(8.62)	32(21.62)	
Secondary (9–10) grade	68(25.75)	38(32.76)	30(20.27)	
Preparatory (11–12)	32(12.12)	30(25.86)	2(1.35)	
College/university	68(25.75)	29(25.00)	39(26.35)	
**Occupation**				
Student	71(26.89)	44(37.93)	27(18.24)	0.98
Unemployed	23(8.71)	4(3.45)	19(12.84)	
Daily labor	29(10.98)	4(3.45)	25(16.89)	
House wife	34(12.87)	8(6.90)	26(17.57)	
Farmer	36(13.63)	8(6.90)	28(18.92)	
Merchant	9(3.40)	6(5.17)	3(2.03)	
Government employee	34(12.87)	30(25.86)	4(2.70)	
Private employer	14(5.30)	8(6.90)	6(4.05)	
Others	14(5.30)	4(3.45)	10(6.76)	
**Monthly income**				
<1000	108(40.90)	29(25.00)	79(53.38)	0.47
1001–3000	58(21.96)	25(21.15)	18(12.16)	
3001–5000	67(25.37)	40(34.48)	10(6.78)	
>5000	31(13.63)	22(18.97)	9(6.08)	

P-value was calculated from chi square test for difference in proportion.

*Prevalence of intestinal parasite*. The overall prevalence of intestinal parasite in this study was 26.13% (95%CI: 21.21% to 31.75]. Of these intestinal protozoa and soil transmitted helminthic infections account 20.45% and 6.06% respectively. Distribution of intestinal parasitic infection by compliance to hand hygiene indicate about 35.13% prevalence in peoples with poor compliance to hand hygiene and 14.65% prevalence among participants with good compliance to hand hygiene.

Age wise distribution of IPIs reviled people aged 15–29 years had a higher prevalence 32.32% (n = 32) than those in other age groups and the lowest prevalence was 16.28% (n = 7) recorded in older aged (>60 years). Gender distribution showed equal prevalence in male and females sex (26.08% vs. 26.19%). Percentage of intestinal parasite positive cases was higher among patients from rural area compared to the urban area (29.54% vs. 24.43%). Similarly high prevalence rate was recorded among illiterate 42.6% (n = 42), agrarian 38.8% (n = 14) and in participants who get less than 1000 ETH Birr.

*Prevalence of intestinal parasitic species*. Altogether eleven different species of intestinal parasites identified where species level prevalence was dominated by *Gardia lamblia* (15.53%), followed by *Entamoeba histolytica/E*. *dispar (*6.44%), and *Ascaris lumbricoides* (1.52%). Prevalence of *Hymenolepis nana*, *Necator americanus or Ancylostoma duodenale* (Hookworm) and *Tricuris trichiura* were 1.14*%* (3/264) each. *Taenia spp*, *and* the nonpathogenic *Entamoeba coli* were detected in 0.76% (2/264) of the study participants. *Strongyloides stercoralis*, *Isosbera beli*, *Cryptosporidium parvum* were detected in single case. Type and prevalence of IPIs varies based on hand hygiene practice category of participants (Table 2).

**Table 2:** Type and frequency of intestinal parasite species identified among patients attending Arba Minch general hospital outpatient department during COVID 19 pandemic, southern Ethiopia (from June to September 2021).

Type of parasites	Total IP	Prevalence by hand hygiene compliance		COR (95% CI)	p-value
		Good (n = 116) N(%)	Poor (n = 148) N (%)		
**Intestinal protozoan species**	62	17(14.66)	45(30.41)	0.38(0.20,0.71)	0.001
*Gardia lamblia*	41	12(10.34)	29(19.59)	0.39(0.18,0.84)	0.008
*E*. *histolytica/E*. *dispar*	17	5(4.31)	12(8.11)		
*Cryptosporidium spp*.	1	0	1(0.68)		
*Isospora belli*	1	0	1(0.68)		
*Entamoeba coli*	2	0	2(1.35)		
**STH**	16	6(4.31)	12(8.11)		
*Ascaris*.*lumbricoides*	4	1(0.86)	3(2.03)		
Hookworm species	3	2(1.72)	1(0.68)		
*Hymenolepis nana*	3	0	3(2.03)		
*Taenia spp*.	2	1(0.86)	1(0.68)		
*Stro*n*gloid stercolaris*	1	0	1(0.68)		
*Tricuris trichiura*	3	1(0.86	3(2.03)		

Abbreviation: STH, soil transmitted helminthes, CI, confidence interval

* statistically significant.

**Fig 1:**
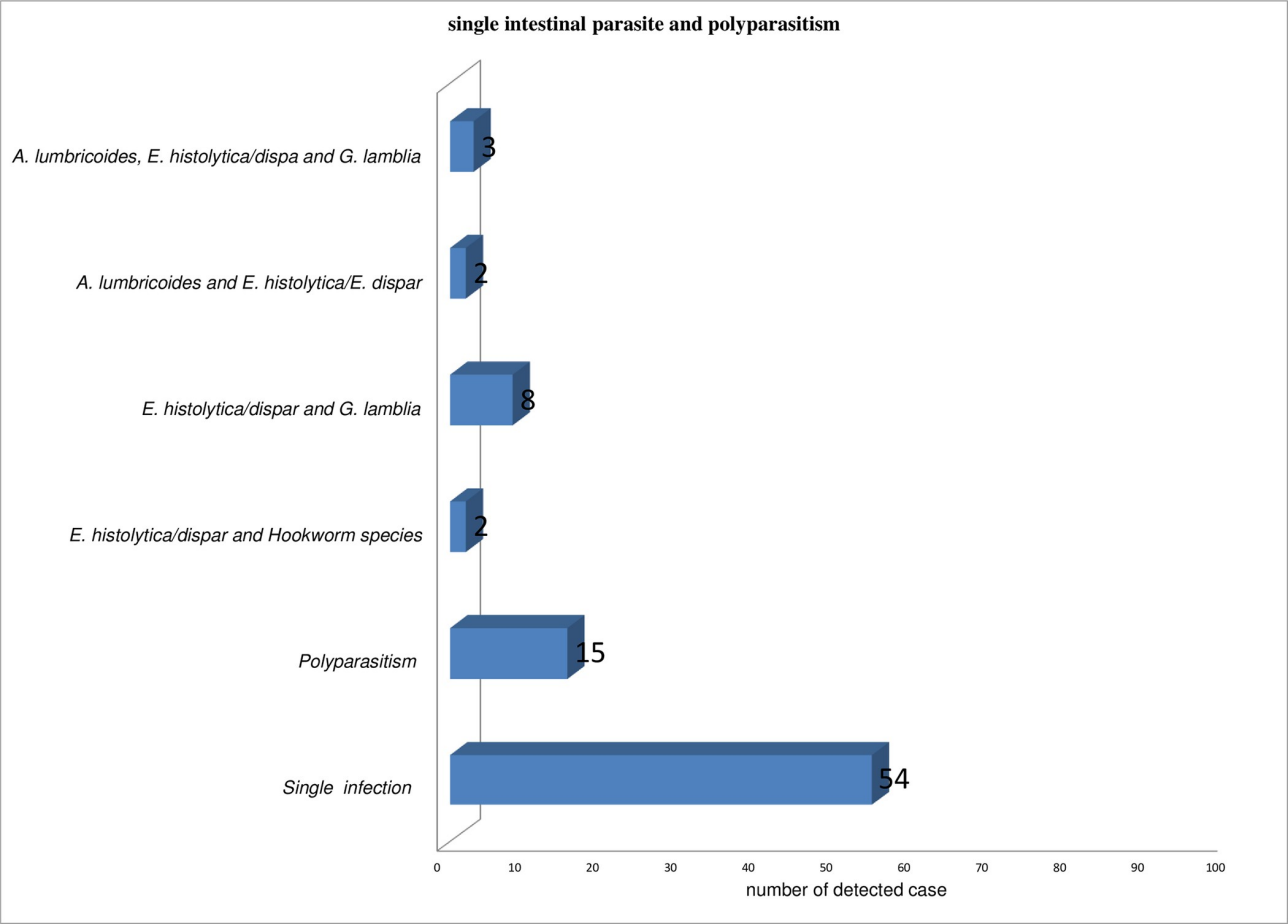
Distribution of single parasitic infection and polyparasitism among patients attending Arba Minch general hospital outpatient department during COVID 19 pandemic (from June-to November 2020). X axis in the bar graph is the type of intestinal parasitic infection and the Y axis is the number of intestinal cases with each type of parasitic infection.

*Prevalence of single parasitic infection and polyparasitism*. This study also noted prevalence of single parasitic infection was 20.45%, (95% CI: 15.6 to 25.3%) whereas 15/69 of cases (5.68%) were polyparasitism i.e. double and triple intestinal parasite infection. Of 15 mixed infection 11 were from poor hand hygiene compliance group. Of this mixed STH and intestinal protozoan co-infections were detected in 4 of 15 of cases. The most common protozoa and helminths co-infection recorded was between *Ascaris lumbricoides and Entamoeba histolytica* (n = 2). The most frequent combination of co-infection was *E*. *histolytica and G*. *lamblia* (8 cases out of 15) and two cases of co-infection with *A*. *lumbricoides* and *E*. *histolytica/E*. *dispar*. In addition, *E*. *histolytica/E*. *dispar*, *G*. *lamblia and A*. *lumbricoides* recorded as triple infections (n = 2). (Fig 1)

*Intensity of infection by species of intestinal helminthes*. The mean egg per gram (epg) of *Ascaris lumbricoides*, *Hookworms* and *Trichuris trichiura* were 488 epg (range: 13 to 1421), 456 epg (range: 8 to 1320) and 300 eggs/g (Range: 11 to 1400), respectively. The overall percentage of light and moderate STH infection was 55.2%, and 7%, respectively. Of which light and moderate infection with *A*. *lumbricoides* was recorded in 72.4%, and 3.3%, for *T*. *trichiura*, and 93.8%, and 3.1% for hookworms (Table 3).

**Table 3:** Intensity of infections ‘with the intestinal nematodes in patients attending Arba Minch general hospital outpatient department during COVID 19 pandemic, southern Ethiopia (from June to September 2021).

Characteristic of STH	Geometric mean (EPG)	Ranges	Intensity of infection Category *		
			Light	Moderate	Heavy
			Percentage	Percentage	Percentage
**Overall intensity of infection**	414	8 to 1421	95.2	4.8	0
**Frequency of light, moderate and heavy infection by species of STH**					
*Ascaris lumbricoides*	688	13 to 2421	3	1	0 (%)
*Hookworm species*	456	8 to 1320	2	1	0 (%)
*Trichuris trichiura*	300	11 to 1400	2	1	0 (%)

** ANOVA P-value < 0.05

Abbreviation: EPG-egg-count per gram.

*Factors associated with intestinal parasite infection*. In the bivariate analysis of the socio-demographic, sanitation and environmental characteristics of the study participants, showed that patients coming from rural areas, age, occupation, monthly income, and compliance to hand hygiene, water source and habit of eating unwashed vegetable associated with overall IPI. However, all socio-demographic characteristics, sanitation issues and environmental related factors with p < 0.25 in bivariate logistic analysis were selected and run for multivariate logistic regression analysis to identify the most important predictors of IPIs.

Younger and adolescent age (15–19), level of education and dietary habit were significantly associated with prevalence of intestinal parasite in the multivariable logistic regression meanwhile regular trimming of figure nail, habit of regular shoe wearing, showed a strong protective effect against intestinal parasites illnesses. Despite habit of treating water, occupation, availability of latrine at home has been selected in the bivariate analysis (p<0.25), they did not remain in the multivariable final model, since they did not reach the level of significance of 5%, previously set for the multivariate analysis. (Table 4).

**Table 4:** Bivariate and multivariate analysis of factors associated with risk of intestinal parasite among patients attending Arba Minch general hospital outpatient department during COVID 19 pandemic, southern Ethiopia (from June to September 2021)(n = 264).

Characteristic	Total tested No.(%)	IPIs (yes) N(%)	Bivariate Analysis		Multivariate Analysis	
			OR (95% CI)	P-value	AOR (95% CI)	P-value
Age categorized (years)						
6–14	13(4.9)	4(30.76)	4.5 (0.84–65.72)	0.57	2.28(1.57,25.8)	0.85
15–29	99(37.5)	32(32.32)	1.5(1.74–13.00)	0.04	2.45(1.21, 8.60)	0.03*
30–44	66(23.86)	19(30.15)	6.1 (7.85–46.38)	0.61	2.22(0.8, 40)	0.77
45–59	46(17.42)	7(15.21)	1.38 (0.64, 3.09)	0.89	1.92(1.0.29)	0.56
>60	43(16.28)	7(16.28)	Ref	Ref	Ref	Ref
Sex of participant						
Male	138(52.3)	36(26.08)	0.88(0.51, 1.51)	0.65		
Female	126 (47.7)	33(26.19)	Ref			
**Highest level of education**						
Illiterate	54(20.45)	23(42.6)	2.64 (1.40, 4.97)	0.002	3.71(1.35, 7.47)	0.03*
1–8 grade	42(15.90)	12(28.57)	1.15 (0.55, 2.41)	0.69	1.09(0.30,3.41)	0.45
9–10 grade	68(25.75)	18(26.47)	1.02 (0.54, 1.91)	0.94	0.96(0.36,3.21)	0.69
11–12 grade	32(12.12)	5(15.62)	0.95 (0.30, 3.03)	0.94	0.63(0.17,4.11)	0.55
Collage and above	68(25.75)	11(17.64	REF	Ref	Ref	
** Residence**						
Urban	176(66.67)	43(24.43)	0.77(0.43, 1.36)	0.373		
Rural	88(33.33)	26(29.54)	Ref			
** Occupation**						
Student	71(26.89) = 49	22(30.98	1.45(0.79, 2.65)	0.224	1.52(0.91, 6.65)	0.30
Unemployed	23(8.71)18	5(21.73)	0.79(0.28, 2.22)	0.659	0.80(0.68, 4.11)	0.65
Daily labor	29(10.98)	8(27.58)	1.08(0.45, 2.58)	0.85	1.28(0.54, 3.58)	0.91
House wife	34(12.87)	10(29.4)	1.20(0.54, 2.67)	0.641	1.73(0.77, 2.65)	0.65
Farmer	36(13.63) = 22	14(38.8)	2.00(0.95, 4.17)	0.064	2.11(0.99,4.21)	0.07
Government employee	34(12.87)	5(14.70)	Ref		Ref	
Others	37(14.01)	5(13.51)	0.41(0.15, 1.115)	0.081	0.81(0.35, 3.25)	0.71
** Finger nail status (Trimmed)**	240(90.90)	58(24.16)	0.58 (0.24–1.37)	0.006	0.60(0.11.0.90)	0.004*
** Finger nail status (Not trimmed)**	24(9.09)	11(45.83	Ref			
** Compliance to Hand Hygiene**						
Good compliance	116(43.93)	17(14..65)	0.31(0.17- 0.58)	0.0003	0.48(0.13,0.68)	0.002*
Poor compliance	148(56.06)	52(35.13)				
** Habit of treating water**						
Yes	51(19.31)	5(9.80)	Ref			
No	213(80.68)	64(30.04)	3.95 (1.50, 10.40)	0.005	3.95(1.4–13.8)	0.35
Habit of Eating raw vegetables or fruits	81(30.68)	36(19.62)	3.95(1.82,5.69)	0.000	3.4(1.87–15.13)	0.012*
Habit of Eating raw vegetables (No)	183(69.31)	36(19.62)	Ref			
Latrine available at home(yes)						
(yes)	235(89.01)	56(23.82)	0.38(0.17, 0.84)	0.018	.43(0.30–8.10)	0.58
(No)	29(10.98)	13(44.82	Ref		Ref	
Protected water source (yes)	210(79.55)	53(52.23)	0.80(0.41–1.55)	0.512		
Protected water source(no)	54(20.45)	16(29.62)	**Ref**			
Regularly wearing Shoe (yes)	180(68.18)	31(17.22)	0.26(0.14, 0.46)	0.000	0.56(0,01,1.23)	0.021

Abbreviations: CI, confidence interval, AOR: adjusted odds ratio:

* p -value less than 0. 05.

*Effect of compliance to hand hygiene rates on intestinal parasitic infections*. The overall proportion of IPIs prevented by good compliance to hand-hygiene during COVID 19 was 52% (95% CI:13% to 68); p = 0.002]. Further analysis of the effect of hand-hygiene practice by critical time adjusted for method of hand hygiene, frequency of hand washing, duration of hand-hygiene and steps of hand-hygiene demonstrated indicate that washing hand before and after meal, hand-hygiene before, during and after handling or preparation of food, hand-hygiene after touching waste or dirty surface, hand-hygiene cleaning child bottom and after visiting sick patients was associated with substantial reduction of intestinal parasitic infection but hand-hygiene after defecation. For example improved hand hygiene before and after meal (adjusted OR: 0.24,(95% CI: 0.16 to 0.88)), followed by before and during handling / preparation of food (adjusted OR:0.33, (95% CI: 0.22, to 0.69)). Hand hygiene after touching and handling any dirty material (adjusted OR:0.58, (95% CI: 0.15 to 0.99)), and after cleaning a child’s bottom or disposing child feces (Adjusted OR: 0.51, (95% CI: 0.01 to 0.73)were also associated with IPIs.

Analysis of the effect of frequency of hand-hygiene, adjusted for critical time, and methods of hand washing/sanitization showed performing hand hygiene for more 6 times a day strongly reduce prevalence rates of intestinal parasitic infection(adjusted OR: 0.34, (95%CI: 0.03 to 0.34)).(Table 5).

**Table 5:** Bivariate and multivariate analysis of hand hygiene practice with risk of intestinal parasitic infection among patients attending Arba Minch general hospital outpatient department during COVID 19 pandemic, southern Ethiopia (from June to September 2021).

Characteristic	Total N (%)	Prevalence of IPIs N (%)	COR(95%CI)	P-value	AOR(95%CI)	P-value
**Critical time for hand hygiene(multiple answers possible)**						
Before &after eating meal (yes	243(92.04)	58(23.86)	0.285(0.111, 0.70)	0.00	0.24(0.16, 0.88)	0.001*
Before &after eating meal(no)	21(7.95)	11(52.38)	Ref			
After defection (toilet)-yes	169(64.01)	36(13.38)	0.71(0.41, 1.21)	0.214	0.68(0.23,0.82)	0.105
After defection (toilet)-(no)	95(35.98)	33((34.73)	Ref			
Before handling and preparing food-(yes)	172(65.00)	32(18.60)	0.34(0.19, 0.59)	0.000	0.31(0.22, 0.69)	0.004
Before handling and preparing food (no)	92 (34.84)	36(39.13)	Ref			
After touching and handling any dirty material (yes)	107(40.00)	16(14.95)	0.36(0.195, 0.68)	0.001	0.58(0.15,0.99)	0.005
After touching and handling any dirty material (no)	157 (59.46)	53(33.75)	Ref			
After cleaning a child’s bottom or disposing child feces(yes)	100(38.00)	12(12.00)	0.25(0.13–0.50)	0.000	0.51(0.01,0.73)	0.004
After cleaning a child’s bottom or disposing child feces(no)	164 (62.12)	56(34.14)	Ref			
**Method of hand washing/ Hand Hygiene**						
Using water	31(13.00)	18(56.25)	Ref		Ref	
Using water and soap	167 (62.00)	15(8.98)	0.87(1.09.3.34)	0.02	0.67(0.16,0.91)	0.005*
Using Water and alcohol-based hand sanitizer	40(15.01)	11(27.00)	1.51(0.91,1.72)	0.78	0.9(0.33,11.0)	0.05*
Using alcohol-based hand sanitizer	26(10.00)	25(26.92)	1.00(0.44,02.19)	0.10*	0.76(0.34, 0.97)	0.31
**Frequency of Hand hygiene per day**						
Always (≥6 times)	100(60.97)	10(10.00)	0.19(0.09, 0.40)	0.000	0.34(03,0.34)	0.002
Sometimes (3–5 times)	74(28.03)	18(24.32)	0.87(0.47, 1.62)	0.675	1.7(1.0,7.76)	0.98
Rarely (≤2 times)	90(34.09)	41(45.56)	Ref	Ref		
**Hand hygiene procedure**						
Demonstrated 6–8 steps	106(40.15)	5(4.71)	0.34(0.22,11.33)	0.98	NA	
Demonstrated 4 to 5 step	58(21.96)	8(13.79)	1.45(0.45,24.06)	0.45	NA	
Demonstrated 4 to 5 steps but in disordered fashioned	60(22.72)	18(30)	2.67(1.56,12.49)	0.88	NA	
Demonstrated less 3 and less key step	40(15.15)	38(95)	Ref	Ref		

Abbreviations: CI, confidence interval, (odds ratio;, * p -value less than 0. 05, IPIs,intestinal parasitic infection, NA, Not applicable.

*Effect of compliance to hand hygiene on intensity of STH infections*. Table 6 illustrates the intensity of STH infection. The overall mean intensity of infection was 414 ova per gram (cpg) of faeces. Mean intensity in those patients who had poor compliance with hand hygiene (403±11egg) was significantly higher (ANOVA (F) = 5.227;(df) = 1; P = 0.023) compared to 211± 10 eggs/g in those who had good compliance to hand hygiene. Analysis of the intensity of infection illustrated, majority of helminthic infection was light (95.2%) followed by moderate infection (4.8%), and no heavy infection. Significantly high proportion of light infection was recorded among patients with good hand hygiene practice compared to poor adherence group.

**Table 6:** Intensity of STH infection by compliance to hand hygiene of patients attending Arbaminch genral hospitals (from June to September 2021).

Intensity Of Infection	Total	Good Compliance Mean + Sd	Poor Compliance Mean + Sd
Mean Intensity Of Infection	414 eggs/g	211± 10eggs/g	403± 11*
Intensity Of STH			
Light	17(95%)	6/6	11 of 12
Moderate	1	0	1
Heavy	0	0	0

* (ANOVA (F) = 5.227;(df) = 1; P = 0.023)

Abbreviation. SD, standard deviations.

## Discussion

Hand hygiene is one of public health intervention suggested to prevent the transmission of IPIs, unfortunately the overall; hand hygiene compliance remains very low to limit the problem. To our knowledge this is the first study to investigate compliance to hand hygiene during acute phase of COVID-19 pandemic and its effect on the prevalence of IPIs and intensity of STH infection.

This study demonstrated that the overall compliance to hand hygiene was 43.93%. It is surprising that the compliance rate observed during an ongoing pandemic, with an increased hand hygiene promotion were similar to observations conducted in pre pandemic years. Overall compliance to hand hygiene of 43.93% suggest shortcoming in the national wide hand hygiene campaign implemented following COVID-19 pandemic. This level of compliance is very low as compared to 90% or more suggested by the world health organization [34]. However, the HH compliance rate reported in this study was also low compared to the 76.4% reported from Japan [35], 94.5% observed by Mieth et al. [36], 79.44% to 96.71% in China [37], 90%- 100% in Australia [38], 60% and 70% reported in recent systemic review [39], 63.45% in European countries [31] and among taxi drivers in Dessie Ethiopia [25]. This result highlights of great concern to the public health campaign on hand hygiene in further control of SARS COV-2 strain. The overall compliance to hand hygiene practice observed in this was similar with other studies [40, 41]. On the other hand, our result reveled higher compliance rate to hand hygiene practice compared to of 14% [26] and 19% [42]. Our experience of higher compliance following national wide HH campaign was similar with other studies that support this hypothesis also reported from different country [29, 43]. In spite of the fact the observed heterogeneity in HH compliance rate in different study may be explained by difference on the study participants, gender difference, HH compliance measurement methods, intervention type and period, availability of water and soap, and broad set of inclusion criteria [37, 40, 44].

The overall prevalence of intestinal parasite obtained in this study was 26.13%(95% of CI: 21.21% to 31.75) and was as high as 52.5% in participants who had poor compliance to hand hygiene practice than good compliance group (14.65%). This clearly demonstrates that IPIs is still a public health problem in the study area where approximately one in four patients was infected with at least one intestinal parasite. The finding was much lower compared to prevalence rate reported from Ethiopia and other developing countries. This includes prevalence of 83% from Jimma [45], 62.3% from Southern Ethiopia [46], 41.3% from Gonder Ethiopia, 62.3% elsewhere [47], 52.9% from northwest Ethiopia [48], 55.0% from Hawassa [5] and 81.0% 37.1% from Chencha Ethiopia [49], 57.1% from Tanzania [50], 34.2% from Iran [51]. Concordant observations were reported from studies conducted in different part of Ethiopia which include prevalence of 20.6% from Jimma Health Center [52]. But the prevalence of intestinal parasite in our study was higher compared to 17.5% prevalence from Brazil [53]. It should be noted that the difference on the prevalence of parasitic infection may be due to geographical area which affects the distribution of parasites, different in socio economic status of peoples, the period at which the study done i.e. seasonal prevalence of parasites especially in relation to before and after COVID-19, laboratory testing method [54]. An alternate and more likely explanation is for this discrepancy also attributed to age, educational status and habit of eating uncooked vegetables in our study population in which both were associated with prevalence of parasitic infection in this study [6].

The present study showed intestinal protozoan infection is common problem in the study are with the prevalence of 23.48% (95% CI: 18.84% to 29.15). This finding agrees with the previous studies that reported higher frequency of protozoan infections than helminthic infections and strongly suggest that transmission related with water [55]. The findings however differ from a study conducted in Ethiopia and surveys conducted in Africa where higher intestinal helminthic infections reported [50, 56, 57]. With regard to the species of intestinal parasites *Giardia lamblia* was found to be the dominant intestinal parasite (15.53 (95% CI: 10.69% to 19.30) followed by *Entamoeba histolytica* /*E*. *dispar* (6.44((95% CI: 3.44 to 9.35. This result ties well with previous studies wherein *Giardia lamblia*, *E*. *histolytica/E*. *dispar* and *A*. *lumbricoides* were reported as the predominant intestinal parasite despite variation in the reported magnitude [54, 56]. Our finding also in agreement with systematic review and meta-analysis which pointed out these two parasites as common causes of intestinal infection [58]. Moving to the helminthic infections it was reported that *Ascaris lumbricoides* was the most common helminth followed by Hookworms *(Ancylostoma duodenale/Necator americanus*, *Hymenolepis nana* and *T*. *trichuria* with the prevalence of 1.52% and 1.14% each, respectively. A similar global ranking of soil transmitted helminths (STHs was obtained by WHO and others [59]. The possible reason for the relatively high prevalence of Ascaris could be their embryonated eggs that have an enormous capacity to withstand environmental extremes and washing with soap and antiseptic hand rub. Furthermore, the eggs are coated with a mucopolysaccharide that renders them adhesive to a variety of surfaces like vegetables, fruits, door handles, and money [6, 60]. The prevalence of the other intestinal helminths (*Taenia spp*., and *S*. *stercolaris*) in the current study was very low and in agreement with priory studies carry out in Ethiopia and neighboring country [47, 52]. The low prevalence might be attributed to the low raw meat consumption during COVID-19 acute phase of pandemic, high shoe wearing habit, good latrine coverage in the geographic area.

It is also interesting to note that the intensity of soil transmitted helminthes infection (*Ascaris lumbricoides*, *Hookworm*, and *T*. *trichuria* in this study was light intensity while the absence of heavily intensity infection suggest the low transmission risk. The finding also confirm the previous conclusion that highlight the low intensity of STH infection in the tropical area [61]. The result enables us to forecast the risk of over dispersion STI soil in the study area is at intermediate since all participants were characterized by nearly similar socio-economic status. However this finding was in disagreement with a report from other part of Ethiopia, where high and moderate intensity was reported [28].

Analysis of factor associated with prevalence of IPIs revealed younger and adolescent age(age group of 15–29 years), illiterate educational status, habit of eating raw vegetable, finger nail status, and compliance to hand hygiene an independent risk factors (Table 4). This finding highlighted the importance of identifying and properly managing socio-demographic, dietary habit and water sanitation and hygiene (WASH) factors that influence the prevalence of intestinal parasite can further decrease IPIs prevalence.

This finding is in agreement with previous similar studies where significantly more IPIs among younger age [62]. But the finding was in contrasts with studies which reported more infection rate among adults than children [55]. This variation may be explained by several reasons; the first reason might be due mass drug treatment administration (MDA) among school children in the previous studies. The second one is that significant number of our study population who had poor hand hygiene practice is the young and adolescent who have ages ranged between 15–29 years old.

Our data confirmed that the peoples with no formal education had 3.71 times (adjusted OR: 3.71, 95% CI: 1.35–7.471, p = 0.038) higher odds of IPIs when compared to those who attended any formal education. This finding confirm the conclusion made in priory study in Ethiopia [63] and the finding suggest the importance of awareness creation to prevent further transmission of IPI among illiterate individuals though this was not addressed in these studies. Analysis of result by fingernail status showed that regular trimming of figure nail was associated with an approximately 40% lower odds of any intestinal parasitic infections (adjusted OR: 0.60 (95%CI: 0.11 to 0.90)). The association between fingernail trimming and intestinal parasitic infections also confirmed finding on the meta-analysis and other study [64, 65]. In the present study participants who eat unwashed raw fruits/vegetables were 3.4 times at higher risk of IPIs compared with those who eat washed raw fruits/vegetables (adjusted OR: 3.4, 95% CI: 1.87 to 15.13, p = 0.004). Similarly, a study conducted in Ethiopia and neighboring country showed habit of eating unwashed fruit or raw vegetable had shown a strong association with parasitic infection [4, 47]. This finding highlights unwashed fruit and vegetables as a potential source of IPIs infection due to the high rate of contamination with Intestinal parasite [66].

As demonstrated by this study, hand hygiene intervention implemented for COVID 19 pandemic had secondary beneficial in significantly reducing the odds of intestinal parasitic infection (AOR = 0.48, (95%CI:0.13 to 0.68),P-value = 0.002 implying that good compliance to hand hygiene can reduce the risk of IPIs by 52%. This study also confirmed that good compliance to hand hygiene had strong protective effect against intestinal protozoa infection (OR:0.38; (95%CI: 0.20 to 0.71), P = 0.001.

The size of effect for intestinal parasitic infection was broadly consistent with previous reviews [15, 44]. This might be explained by good hand hygiene practice broadly removes any pathogens from contaminated hand mechanically and may also chemically kill pathogens rendering them non infective ultimately reduce the feco-orally transmitting IPIs [44, 67]. The finding was in agreement with a 48% of reduction of IPIs and other infection in the existing literature and systematic review [22, 40]. Moreover the protective effect of hand hygiene attained in the present study is consistent with results of similar studies in Kenya and trials in Karachi, Pakistan [68]. Similarly magnitude of reductions was estimated in a recent systemic review [44]. Good compliance to hand hygiene had no significant effect on prevalence of STH infection on this study. The finding may be explained by the route of parasitic transmission other than feaco oral route such as larval skin penetration contaminated raw vegetables, untrimmed finger nail, or limited changes in hand washing behavior [16].

In sub group analysis the effect of hand hygiene intervention was only significantly associated with decrease rate of intestinal protozoan disease risk (p = 0.003) and *Gardia lamblia* but not with STH infection. This was similar finding with study in Kenya [69].However, none of the results of as general and other species of IPI as specific were significantly associated with compliance to hand hygiene practice. The lack of protective effect might be due to small number of detected parasite.

## Conclusion

Compliances to hand hygiene recommendation during COVID-19 appears notably low than the WHO target, This calls for smeasure to improve adherence to this broad infection control measure is yet a major concern in general community.

This study also confirmed compliance to hand hygiene during COVID -19 was associated with significant reduction in odds of IPIs by demonstrating compliance rate of 43.93% result in 52% of reduction in prevalence of IPIs. Moreover compliance to hand hygiene showed protective effect against intestinal protozoa. Differences in the critical time of hand hygiene and method (using soap and water) found to account for the protective effect of HH against IPIs. In general, the findings in this study highlighted the concept that HH are an effective general infection control measure and/or augment the existing intestinal parasitic infection control tools, provided that compliance to hand hygiene was good. Use of soap and water should be the method of choice for hand washing.

Our result also suggest a prevalence of IPIs in the study area indicating intestinal parasitic infections yet considerable public health problems where infections were dominated by intestinal protozoan (*G*. *lamblia*, *E*. *histolytica*) and prevalence of IPIs were associated with younger age, illiterates, finger nails status, and habit of consuming raw vegetables. Therefore, our study emphasizes and offer useful insight for public health expertise/clinician/policy makers, and researchers to conduct further carefully crafted study covering longer period of time in order to confirm the observed reduction in the rate and intensity of intestinal parasite. Implement program that promote and monitor HH to sustain the current level of improvement, to retain rigorous hand hygiene as a cost effective and efficacious process for intestinal parasitic infections.

### Limitations of the study

This study has subjected to the following limitations: first, we expected recall bias because we collected information about the hand-hygiene practice from self-reported questionnaire. Managing this limitation was by carefully training the data collectors. Occurrence of IPIs was calculated from examination of single stool sample and this may underestimate the true picture outcome. However the study used wet mount, concentration and staining methods to come up these limitations. The other limitation was due to resource constraints, we did not perform molecular techniques like PCR to identify and discriminate the true pathogenic *E*. *histolytica* from *E*. *dispar* given that the diagnostic method we used (FECT) does not allow the distinction between pathogenic *E*. *histolytica* from non-pathogenic *E*. *dispar*. Thus, we can’t rule out the possibility of over diagnosis of amoebiasis. The study was conducted in single instruction and used cross sectional design; therefore the evidence may not applicable to policy and practice because the evidence has very limited generalizability. Thus we recognize the low sensitivity of this methodology if compared with randomized controlled trials (RCTs), cluster RCTs, quasi‐RCTs (qRCTs), and controlled before‐and‐after studies. Therefore future studies should take into account these limitations.

## Supporting information

S1 Table A summary of the questions and the scale used for measurement. (DOCX)

S2 Table Characteristics of study participants stratified by socio-demographic, water and sanitation and hand hygiene practice. (DOCX)

S1 Data (XLSX)

## Acronyms/abbreviations

AMU: Arba Minch University
CDC: Centers for Disease Control and Prevention
CI: Confidence interval
HH: hand hygiene
NTD: neglected tropical disease
STH: soil transmitted helminthes
COD: crude odds ratio
AOR: adjusted odds ratio
IPIs: Intestinal parasitic infections
COVID -19: corona virus disuse
MDA: mass drug administration
WASH: water sanitation and hygiene
SPSS: social science statistical software
SD: standard deviation
WHO: World Health Organization
EPG: eggs per gram of stool
