# Supplementary material for: The effect of compliance to Hand hygiene during COVID-19 on intestinal parasitic infection and intensity of soil transmitted helminthes, among patients attending general hospital, southern Ethiopia: Observational study
Source: PLoS One. 2022 Jun 29;17(6):e0270378. doi: 10.1371/journal.pone.0270378 (PMC9242515; doi:10.1371/journal.pone.0270378)
Supplement: S1 Table — (DOCX) [file pone.0270378.s001.docx]

# Questionnaires/English version

**Title:** The effect of compliance to Hand hygiene during COVID-19 on intestinal parasitic infection and intensity of soil transmitted helminthes among patients attending general hospital, southern Ethiopia: Observational study

**Code number of the participant ____________ date:-_______________**

**Data collector name _______________ signature __________________**

**PART I: questionnaire to collect socio demographic and WASH related data**

| **S,NO** | **QUESTION** | **CATEGORY/code** | | **REMARK** | |
| --- | --- | --- | --- | --- | --- |
| **Socio-demographic characteristics of study subjects** | | | |  |  |
| 1 | Sex | | 1. Male 2. Female |  | |
| 2 | How old are you? | | ____________years |  | |
| 3 | Residence | | 1. Urban 2. Rural |  | |
| 4 | What is your occupation? | | 1. Student 2. Unemployed 3. Daily labor 4. House wife 5. Farmer 6. Merchant 7. Government employee 8. Others(specify)_________ |  | |
| 5 | Educational status | | 1. Illiterate 2. Primary (1-8) 3. Secondary (9-10) 4. Preparatory(11-12) 5. Higher education |  | |
| 6 | Monthly income( in Birr) | | __________Ethiopian birr |  | |
| 1 | Water source for drink&preparation of food? | | 1. Pipe  2. River  3. Spring  4. Well |  | |
| 2 | Do you have latrine availability? | | A. Yes  B. No |  | |
|  | If no,where you defecate? | | 1.Open field  2.Neighbour  3.Public  4.Other |  | |
| 4 | Trimming status of finger? | | A.Trimmed  B. Untrimmed |  | |
|  | If yes | | 1. Always  2. Sometimes  3. Not at all |  |  |
| 5 | Do you have habit of eating raw meat wthin last 2 month? | | 1. Yes  2. No |  | |
| 6 | Do you have habit of  eating raw vegitable | | A. Yes  B. No |  | |
| 7 | Do you treat water for drinking? | | 1.Yes  2.No |  | |

**Part II: compliance to hand hygiene**

**Checklist to assessment of compliance to hand hygiene**

| Hand washing at critical time | Measurement Scale | | | | |
| --- | --- | --- | --- | --- | --- |
| Do you wash/sanitize your hand before/after of meal | 1 | 3 | 3 | 4 | 5 |
| Do you wash/sanitize your hand after toilet |  |  |  |  |  |
| Do you wash/sanitize your hand before/while preparing food |  |  |  |  |  |
| Do you wash/sanitize your hand after touching and handling any dirty material |  |  |  |  |  |
| Do you wash/sanitize your after cleaning a child’s bottom or disposing child feces |  |  |  |  |  |
| **Method of hand washing/** **Hand Hygiene (multiple answers possible** |  |  |  |  |  |
| How often do you use only water for washing hand |  |  |  |  |  |
| How often do you use water and soap |  |  |  |  |  |
| How often do you use Water and alcohol-based hand sanitizer |  |  |  |  |  |
| How often do you use only alcohol-based hand sanitizer |  |  |  |  |  |
| **Frequency of Hand hygiene per day** |  |  |  |  |  |
| Do you wash your hands ≥6 times per day |  |  |  |  |  |
| Do you wash your hand 3–5 times |  |  |  |  |  |
| Do you wash your hand ≤2 times |  |  |  |  |  |
| **For How long wash hand for each session(duration)** |  |  |  |  |  |
| Greater than 1 min |  |  |  |  |  |
| 15-to 30 second |  |  |  |  |  |
| >15 second |  |  |  |  |  |
| Procedure |  |  |  |  |  |
| Demonstrate the steps of hand washing you practice daily |  |  |  |  |  |
| Demonstrate the steps of hand sanitization |  |  |  |  |  |

**Supervisor name _______________________ signature ______________________**
