# Supplementary material for: The effect of compliance to Hand hygiene during COVID-19 on intestinal parasitic infection and intensity of soil transmitted helminthes, among patients attending general hospital, southern Ethiopia: Observational study
Source: PLoS One. 2022 Jun 29;17(6):e0270378. doi: 10.1371/journal.pone.0270378 (PMC9242515; doi:10.1371/journal.pone.0270378)
Supplement: S2 Table — (DOCX) [file pone.0270378.s002.docx]

**S2 Table . Characteristics of study participants stratified by Socio-demographic, water and sanitation and hand hygiene practice in Arba Minch town, southern Ethiopia between (from June to September 2021)(n= 264)**

| Variables | Frequency(%) |
| --- | --- |
| Age(years) |  |
| 6- 14 | 13(4.92) |
| 15-29 | 99(37.50) |
| 30-44 | 63(23.86) |
| 45-59 | 46(17.42) |
| >60 | 43(16.28) |
| Sex | |
| Male | 139(52.30) |
| Female | 125(47.34) |
| Residence |  |
| Urban | 176(66.67) |
| Rural | 88(33.33) |
| Educational status |  |
| Illiterate/read and write | 54(20.45) |
| Primary (grade1-8)grade | 42(15.90) |
| Secondary (9-10) grade | 68(25.75) |
| Preparatory (11-12 ) | 32(12.12) |
| College/university | 68(25.75) |
| Occupation |  |
| Student | 71(26.89) |
| Unemployed | 23(8.71) |
| Daily labor | 29(10.98) |
| House wife | 34(12.87) |
| Farmer | 36(13.63) |
| Merchant | 9(3.40) |
| Government employee | 34(12.87) |
| Private employer | 14(5.30) |
| Others | 14(5.30) |
| Monthly income |  |
| <1000 | 108(40.90) |
| 1001-3000 | 58(21.96) |
| 3001-5000 | 67(25.37) |
| >5000 | 31(13.63) |

Table

| **WASH** |  |
| --- | --- |
| Finger nail status (trimmed) | 240(90.9) |
| Pipe(tape) | 210(79.55) |
| River | 17(6.44) |
| Well | 11(4.17) |
| Multiple source | 26(9.85) |
| Habit of Treating drinking water(yes) | 51(19.31) |
| Habit of Eating raw vegetables or unwashed fruits (yes) | 81(30.68) |
| Habit of Eating raw meat (yes) | 119(45.07) |
| Habit of Wearing shoe (yes) | 246(93.70) |
| Availability of Latrine at home (yes) | 235(89.01) |
| Frequently using latrine use (yes) | 161(61.00) |
| **HAND Hygiene practice** |  |
| Hand Hygiene at critical times (multiple answers possible ) | |
| Before &after eating meal | 243(92.04) |
| After defection (toilet) | 169(64.01) |
| Before handling and preparing food | 172(65.00) |
| After touching and handling any dirty material (yes | 107(40.00) |
| After cleaning a child’s bottom or disposing child feces(yes ) | 100(38.00) |
| How do you wash your hands? (multiple answers possible ) |  |
| With water | 31(13.00) |
| With water and soap | 167(62.00) |
| With Water and alcohol-based hand sanitizer | 40(15.01) |
| With alcohol-based hand sanitizer | 26(10.00) |
| Frequency |  |
| Always (≥6 times ) | 10(10.00) |
| Sometimes (3–5 times) | 18(24.32) |
| Rarely (≤2 times) | 41(45.56) |
| **Hand hygiene procedure** |  |
| Demonstrated 6-8 steps | 106(40.15) |
| Demonstrated 4 to 5 step | 58(21.96) |
| Demonstrated 4 to 5 steps but in disordered fashioned | 60(22.72) |
| Demonstrated less 3 and less key step | 40(15.15) |
